# Scurvy in an autistic child: early disease on MRI and bone scintigraphy can mimic an infiltrative process

**DOI:** 10.1259/bjrcr.20150148

**Published:** 2015-07-07

**Authors:** N Khan, J M Furlong-Dillard, R F Buchman

**Affiliations:** ^1^ Royal Stoke University Hospital, University Hospitals of North Midlands NHS Trust, Stoke-on-Trent, UK; ^2^ Department of Pediatric Radiology, Department of Pediatrics, University of Arkansas for Medical Sciences, Arkansas Children’s Hospital, Little Rock, AR, USA

## Abstract

Scurvy is uncommon in the industrialized world and is rarely reported in the paediatric population. Children with developmental and neuropsychiatric disorders and poor oral intake are at increased risk. The classic appearance of scurvy on radiographs is well documented. However, in early disease, radiographs may be normal. Bone scintigraphy can detect early disease, but involves radiation and findings are usually non-specific. MRI can detect very early disease in patients with scurvy prior to radiological findings and does not involve radiation. We present a case of unsuspected scurvy in an autistic child who had abnormality confined to the metaphysis seen on both MRI and bone scintigraphy. Early diagnosis and treatment in our patient prevented more serious complications such as fractures and subperiosteal haemorrhages.

## Introduction

Scurvy is a disease caused by deficiency of vitamin C. It is commonly seen in countries with low socioeconomic status and is rare in well-developed countries.^1^ In industrialized countries, most cases occur in the elderly and alcoholics. Scurvy in children is rare but has been reported in individuals with developmental and neuropsychiatric disorders, leading to difficulty maintaining adequate nutrition.^1^ Children with scurvy can present with variable symptoms such as sudden onset of difficulty walking, which has a wide differential diagnosis.^1^ Symptoms can be missed in mentally challenged children, making the diagnosis of scurvy a challenging one.^1,2^

Scurvy on conventional radiographs has a classic appearance and has been well described in the medical literature. Early disease detection with radiographs is limited and radiographs may appear normal. MRI and bone scintigraphy are both more sensitive in detecting bone marrow pathology compared with conventional radiographs, but there is little in the literature describing the appearance of scurvy with these two imaging modalities.^1–6^ We present an unusual case of unsuspected scurvy in an 8-year-old autistic male whose abnormal findings on MRI and bone scintigraphy have not previously been described.

## Case report

**Figure 1.**
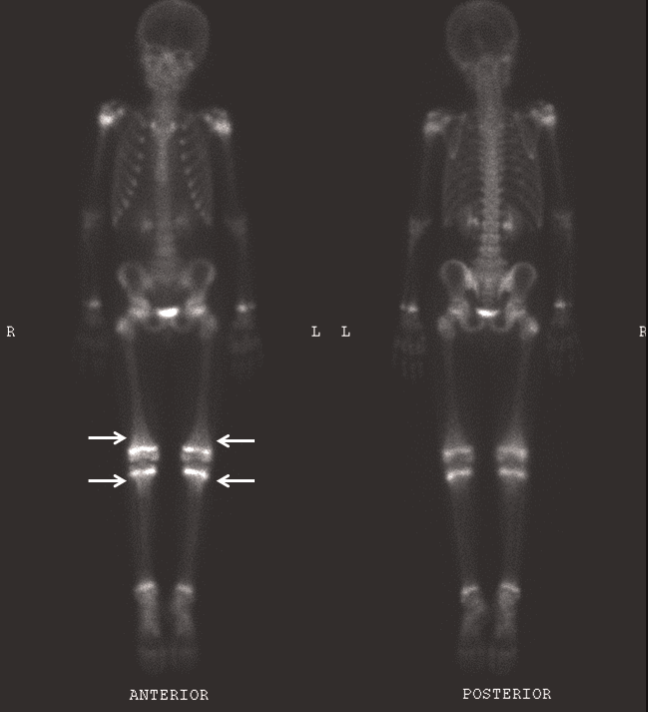
Whole-body bone scintigraphy demonstrates increased radiotracer activity in bilateral shoulders, wrists, hips, knees and ankles, most severe in the knees (*arrows*).

**Figure 2.**
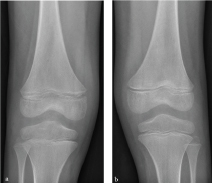
(a) Right and (b) left anteroposterior radiographs of the knees were normal. Radiograph of bilateral knees and pelvis was normal (not shown).

**Figure 3.**
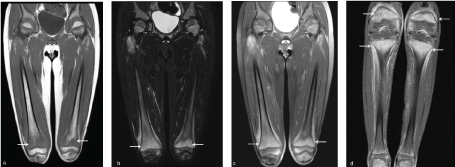
(a) Coronal *T*_1_ and (b) short tau inversion-recovery (STIR) images demonstrate patchy marrow and subperiosteal signal abnormality hypointense on *T*_1_ and hyperintense on STIR in bilateral distal femoral metaphysis *(arrows*). (c) Bilateral femurs and (d) tibia/fibula post contrast-enhanced fat-suppressed coronal *T*_1_ images demonstrate intense subperiosteal and metaphyseal marrow enhancement in bilateral distal femurs and proximal tibiae (*arrows*).

An 8-year-old autistic male presented to the emergency department with severe bilateral leg pain and difficulty walking. Additional recent medical history included gum swelling and bleeding, low-grade fever and a maculopapular rash in bilateral upper and lower extremities. Routine blood work, additional tick titres and autoimmune workup were all normal. A clinical diagnosis was unclear and a whole-body bone scintigraphy examination was ordered followed by subsequent radiographs. Bone scintigraphy demonstrated increased radiotracer activity in bilateral shoulders, wrists, hips, knees and ankles, most severe in the knees (Figure 1). Radiographs of the above-mentioned areas were all normal (Figure 2). Differential considerations included infiltrative processes such as leukaemia, neuroblastoma metastases and multifocal osteomyelitis. Multifocal fractures were felt to be unlikely. Further evaluation with MRI was recommended. Subsequent contrast-enhanced MRI of both femurs demonstrated intense metaphyseal signal abnormality and enhancement in bilateral proximal and distal femurs and proximal tibiae (Figure 3). Subperiosteal signal abnormality and enhancement along the metaphysis of both femurs and tibiae was also observed (Figure 3). MRI findings correlated with findings seen on whole-body bone scintigraphy but were occult on radiographs. An infiltrative process such as leukaemia was of primary concern.

Following MRI, a peripheral blood smear and a bone marrow aspiration were obtained to evaluate for haematological malignancy; both were negative. Urine and blood cultures were obtained and both were normal. Perplexed by the abnormal imaging findings and normal laboratory work-up, further discussion with the patient’s mother revealed that the patient’s diet consisted solely of cookies, brown sugar pop tarts, chocolate milk and Krispy Kreme doughnuts. Nutritional deficiency was considered the cause of the patient’s symptoms and a complete vitamin panel was ordered that revealed a low vitamin C level of 0.1 mg dl-^–1^ (normal 0.4–2.0 mg dl^–1^). All other vitamins were normal. A clinical diagnosis of vitamin C deficiency or scurvy was established.

**Figure 4.**
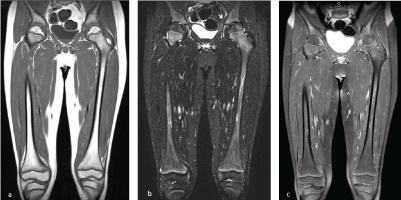
(a) Coronal *T*_1_, (b) coronal short tau inversion-recovery and (c) contrast-enhanced fat-suppressed coronal *T*_1_ images obtained after 8-month treatment with supplemental vitamin C therapy demonstrate normal marrow signal and enhancement throughout both femurs.

Treatment consisted of corrective nutritional measures and supplemental vitamin C therapy. The patient’s symptoms rapidly improved and he was discharged home and instructed to take 100 mg of supplemental vitamin C twice a day. After approximately 8 months of supplemental vitamin C therapy, the patient returned for a follow-up MRI of both femurs. Metaphyseal abnormalities seen on initial MRI had completely resolved on the follow-up MRI examination (Figure 4).

## Discussion

Vitamin C or ascorbic acid is an essential vitamin. It is water-soluble, cannot be stored in the body for long periods of time and has to be replaced daily. Deficiency of this vitamin results in the development of scurvy that occurs after 1–3 months of poor vitamin ingestion below 10 mg dl-–1.^1^ Patients typically demonstrate fatigue, failure to gain weight, loss of appetite and irritability. Vitamin C forms an important protein that aids in the formation of skin, tendons, ligaments and blood vessels. Its deficiency causes fragility of small blood vessels and capillaries resulting in petechiae, ecchymosis and gingival hypertrophy.^1^ Vitamin C also contributes to the synthesis of collagen, which is needed for the repair and maintenance of cartilage, bones and teeth.^1,2^ Poor collagen formation weakens bone, leading to microfractures, subperiosteal haemorrhage and pain and swelling.^1–4^ Musculoskeletal symptoms, including bone pain and difficulty walking, affect 80% of patients with scurvy.^5–7^

Scurvy is mainly seen in countries with poor socioeconomic status and is uncommon in the well-developed world. Alcoholics and elderly individuals living alone have a higher incidence of developing scurvy.^5^ In industrialized nations, scurvy is particularly rare in the paediatric population but can be seen in children with poor nutrition. Most documented cases of paediatric scurvy involve children who are autistic, have cerebral palsy or have some other developmental or neuropsychiatric disorder in whom a healthy nutritional status may be difficult to maintain.^1–6^

Patients with autism prefer foods with a smooth texture and a high glycaemic index, as was the case with our patient.^2^ Paediatric scurvy may also be seen in infants who are fed evaporated or boiled milk in which ascorbic acid is destroyed by heat.^1,7^

The classic appearance of scurvy on radiographs is well described in the medical literature and listed in Table 1.^1–5^ Findings on radiographs usually appear after 3–6 months of vitamin C deficiency.^1–4^ Initial imaging usually begins with radiographs of the upper and lower extremities. With early disease, most patients have normal radiographs or findings of diffuse demineralization.^1–3^ Radiographs of bilateral arms and legs in our patient were all normal, indicating early disease.

**Table 1.** Classic radiographic signs of scurvy.

Specific signs	Radiographic description
Wimberger’s ring	Small lucent epiphysis surrounded by a sharp sclerotic rim
Frankel’s line	Dense zone of provisional calcification in the metaphysis
Trummerfeld zone	Lucent metaphyseal band underlying Frankel's line
Pelkan’s spur	Metaphyseal spur that results in cupping of the metaphysis
Subperiosteal haemorrhages	Areas of increased density along the bones that eventually develop rim of calcification

MRI is a highly sensitive imaging modality for the evaluation of subtle changes in water content within bone marrow, not seen with radiographs. It is likely that acute and early changes of vitamin C deficiency may only be seen with MRI. There are a small handful of case reports that describe MRI findings in scurvy.^1–6^ The most common findings described on MRI include focal or diffuse marrow signal abnormality and enhancement.^1–6^ It is unclear if the bone marrow changes on MRI are due to oedema, microfractures, haemorrhage or a combination of these.^2–4^ Subperiosteal haemorrhages can appear heterogeneous on MRI with associated oedematous changes in surrounding tissue.^4,6^ The appearance of blood products in the subperiosteal fluid collection should raise the suspicion of scurvy. Our case did not demonstrate evidence of subperiosteal haemorrhage, but there are short tau inversion-recovery hyperintensity and enhancement involving the periosteum along the distal metaphysis of the femurs bilaterally. This was also seen in another published case report.^2^ Most of the reported MRI findings with scurvy also had accompanying classic radiographic findings involving the long bones.^1,2,6^ Band-like signal abnormality confined to metaphysis has been reported once previously and biopsy demonstrated gelatinous material.^3^ Gelatinous transformation indicates atrophic changes of bone that can be seen in a variety of conditions such as chronic renal insufficiency, anorexia, tuberculosis, cancer-related cachexia and HIV.^3^ The metaphysis being a site of rapid turnover may be affected first in patients with acute vitamin C deficiency due to poor collagen formation needed for bone growth and repair.

Bone scintigraphy is another imaging modality that can detect early changes in bone marrow; however, findings with bone scintigraphy suffer from low specificity. The appearance of scurvy with bone scintigraphy is less well known than with MRI.^6–8^ Bone scintigraphy findings in patients with scurvy can be normal despite abnormalities on MRI and radiographs.^1,4^ Patchy or diffuse uptake in the long bones that may be unilateral or bilateral and symmetric in pattern has been described.^6–8^ Uptake can also be seen along the vascular periosteum from subperiosteal reaction in early disease and late scurvy when subperiosteal haemorrhage calcifies.^8^ The subperiosteal reaction and the calcified haematoma in the diaphysis can give a club-shaped appearance in bone scintigraphy.^7,8^ There may also be uptake in the surrounding soft tissues and reduced uptake in growth centres.^6^ All cases described in the medical literature had associated abnormalities on radiographs, which suggests advanced disease. Our patient demonstrated radiotracer uptake confined to the metaphyseal regions of long bones. To our knowledge, this pattern has not been previously described in a patient with scurvy. It is likely that the bone scintigraphy findings in our case represent an acute or early phase of vitamin C deficiency, before any radiographic abnormalities could develop.

In summary, we present an unusual case of unsuspected paediatric scurvy. Our initial differential included an infiltrative process such as leukaemia, neuroblastoma metastases and multifocal osteomyelitis. Differential considerations in a patient with isolated metaphyseal abnormality on bone scintigraphy and MRI should include scurvy, particularly in patients with poor nutrition and developmental or neuropsychiatric disorders. These findings likely reflect an early stage of scurvy disease and early diagnosis and treatment can prevent more serious complications such as fractures and subperiosteal haemorrhages.

## Learning points

1. Scurvy should be considered in autistic children who present with bone pain and have a preference for foods with a high glycaemic index.
2. The early findings of scurvy on MRI and bone scintigraphy can mimic an infiltrative process.

## Ethics approved

The case report meets the institutional requirements of the ethics committee.
